# RIOK3 promotes mTORC1 activation by facilitating SLC7A2-mediated arginine uptake in pancreatic ductal adenocarcinoma

**DOI:** 10.18632/aging.204528

**Published:** 2023-02-24

**Authors:** Henan Qin, Rui Sun, Xin Guo, Lei Fang, Mengyuan Xu, Yibin Teng, Ning Zhen, Aman Wang, Jiwei Liu

**Affiliations:** 1 The First Affiliated Hospital of Dalian Medical University, Dalian 116000, China; 2 Hangzhou Medical College Affiliated Lin An People’s Hospital, Hangzhou 310000, China; 3 Liaoning Key Laboratory of Molecular Targeted Drugs in Hepatobiliary and Pancreatic Cancer, Dalian 116000, China

**Keywords:** RIOK3, SLC7A2, arginine metabolism, pancreatic ductal adenocarcinoma, Treg

## Abstract

Pancreatic ductal adenocarcinoma (PDAC) is a highly aggressive malignancy with a poor prognosis. Reprogramming of amino acid metabolism is one of the characteristics of PDAC, in which arginine metabolism is significantly altered in PDAC cells and is involved in important signaling pathways. Current studies have identified arginine deprivation as a potential strategy for PDAC treatment. In this study, we performed Liquid Chromatograph Mass Spectrometer (LC-MS)-based non-targeted metabolomic analysis on PDAC cell lines with stable Rio Kinase 3 (RIOK3) knockdown and PDAC tissues with different RIOK3 expressions and found that RIOK3 expression was significantly correlated with arginine metabolism in PDAC. Subsequent RNA sequencing (RNA-Seq) and Western blot analysis showed that RIOK3 knockdown significantly inhibited the expression of arginine transporter solute carrier family 7 member 2 (SLC7A2). Further studies revealed that RIOK3 promoted arginine uptake, mechanistic target of rapamycin complex 1 (mTORC1) activation, cell invasion, and metastasis in PDAC cells via SLC7A2. Finally, we found that patients with high expression of both RIOK3 and infiltrating Treg cells had a worse prognosis. Overall, our study found that RIOK3 in PDAC cells promotes arginine uptake and mTORC1 activation through upregulation of SLC7A2 expression, and also provides a new therapeutic target for therapeutic strategies targeting arginine metabolism.

## INTRODUCTION

Pancreatic ductal adenocarcinoma (PDAC) is one of the most common tumors of high malignancy. The 2020 World Health Organization (WHO) GLOBOCAN project estimates that there will be more than 490,000 new cases of pancreatic cancer and more than 460,000 new deaths worldwide in a year, and the mortality to incidence ratio (M/I) is about 0.94, ranking first among all common tumor species [1, 2]. Currently, the overall 5-year survival rate for PDAC is only about 7%, and it is estimated that by 2030, PDAC deaths will be the 2nd leading cause of tumor-related death in the United States after non-small cell lung cancer [3]. Surgical resection is the only effective treatment for PDAC, and chemotherapy remains the first choice for patients with unresectable PDAC [4]. However, multiple treatments such as chemotherapy, radiotherapy, immunotherapy, and targeted therapy have not significantly improved the long-term survival rate of PDAC patients [5–7]. Therefore, a breakthrough is still needed in the treatment of PDAC.

Metabolic reprogramming is an important feature of tumors [8]. In addition to glucose and lipid metabolic reprogramming, abnormal amino acid metabolism also plays an important role in the rapid growth and proliferation of cancer cells [9–11]. Arginine is a non-essential or semi-essential amino acid that is involved in biological processes such as cell proliferation, survival, and protein synthesis. It is also the precursor of nitric oxide (NO), polyamine, proline, creatine, glutamate, and other metabolites [12]. Importantly, the mechanistic target of rapamycin complex 1 (mTORC1) can be activated by arginine sensing, which in turn releases essential amino acids from lysosomes and promotes PDAC cell growth. In contrast, arginine deprivation significantly inhibited the mTORC1 activation [13–16]. Current studies have found that arginine deprivation significantly inhibits the growth of PDAC cells with low Argininosuccinate synthase (ASS1) expression [17]. In addition, arginine deprivation promotes the sensitivity of PDAC to chemotherapy, radiotherapy, and other therapies [18, 19]. Therefore, the development of new therapeutic targets targeting arginine metabolism is expected to provide new options for the treatment of PDAC.

In this study, we found that Rio Kinase 3 (RIOK3) was significantly correlated with arginine metabolism in PDAC by Liquid Chromatograph Mass Spectrometer (LC-MS)-based untargeted metabolomics analysis. High-throughput transcriptome analysis showed that RIOK3 knockdown significantly inhibited the expression of arginine transporter solute carrier family 7 member 2 (SLC7A2). Further studies showed that RIOK3 promoted arginine uptake, mechanistic target of rapamycin complex 1 (mTORC1) activation, cell invasion, and metastasis through SLC7A2 in PDAC cells. Finally, we found that the RIOK3 in PDAC tissues was significantly positively correlated with the number of infiltrating Regulatory T (Treg) cells, and the prognosis of PDAC patients with high expression of both RIOK3 and infiltrating Treg cells were worse. Our study revealed that RIOK3 is one of the kinesins responsible for arginine uptake by PDAC cells, which also provides a new therapeutic target for therapeutic strategies targeting arginine metabolism.

## RESULTS

### RIOK3 protein is highly expressed in PDAC tissues and associated with poor prognosis

**Figure 1:**
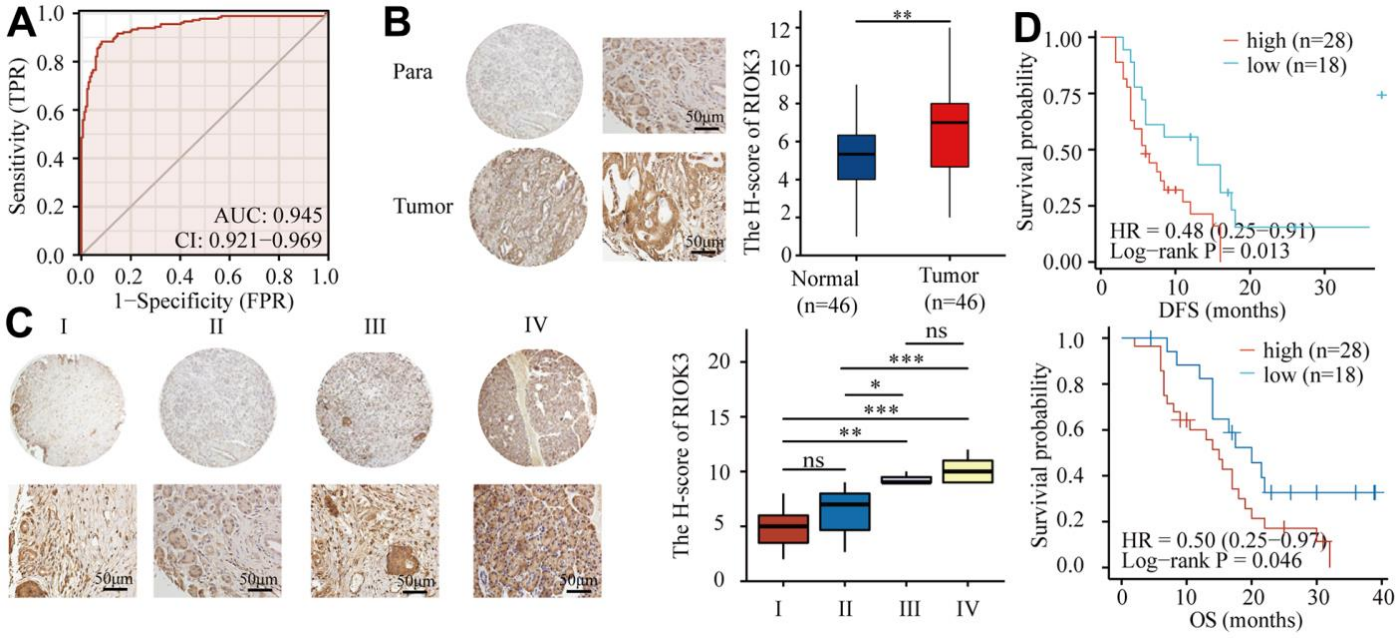
**RIOK3 protein is highly expressed in PDAC tissues and associated with poor prognosis.** (**A**) ROC curve of RIOK3 mRNA for prediction PDAC in TCGA and GTEx datasets (AUC=0.954, 95%CI:0.921-0.969) (n=350, 171 Normal and 179 Tumor). (**B**) Representative IHC staining of RIOK3 and IHC analysis of RIOK3 expression in PDAC and paired adjacent normal tissue (n=46; p=0.004). Paired samples Wilcoxon test. (**C**) Representative IHC staining of RIOK3 and IHC Immunohistochemical analysis of RIOK3 expression in different stages of PDAC tissues (n=46; 15 Stage I, 21 Stage II, 3 Stage III, and 7 Stage IV; F = 17.331, P < 0.001). One-way ANOVA test. (**D**) Kaplan–Meier curves of DFS and OS analysis of RIOK3 expression in PDAC patients (n=46, 28 high-RIOK3 and 18 low-RIOK3). The star (*) symbol denotes the level of statistical significance: * p < 0.05, ** p < 0.01, *** p < 0.001, ns no significance.

In our previous study, bioinformatics analysis of PDAC transcriptome data derived from The Cancer Genome Atlas (TCGA) and the Genotype-Tissue Expression (GTEx) database revealed that RIOK3 mRNA was significantly overexpressed in PDAC tissues [20]. In this study, we used these data to perform Receiver operating characteristic (ROC) analysis and showed that RIOK3 mRNA expression had a good predictive ability to distinguish PDAC tissues from normal pancreatic tissues. The area under curve (AUC) was 0.945 (95% confidence interval [CI] = 0.921-0.969) (Figure 1A). To further clarify the protein expression of RIOK3 in PDAC tissues, we performed immunohistochemical (IHC) analysis on a tissue microarray containing 46 pairs of PDAC and adjacent normal tissues and found that the protein expression (H-score) of RIOK3 in PDAC tissues was significantly higher than that in adjacent normal tissues (Figure 1B). 46 PDAC patients‘ baseline characteristics were listed in Table 1. Interestingly, similar to previous findings, the protein expression of RIOK3 was abnormally upregulated as the pathological stage increased (Figure 1C). Furthermore, patients with high RIOK3 expression had a significantly shorter overall survival (OS) (20.0 vs. 15.0 months, HR = 0.50, 95% CI 0.25-0.97, p = 0.046) and disease-free survival (DFS) (13.0 vs. 6.0 months, HR = 0.48, 95% CI 0.25-0.91, p = 0.013) than those with low expression (Figure 1D). Overall, our study found that the RIOK3 protein was significantly overexpressed in PDAC tissues and was associated with a poor prognosis.

**Table 1:** Baseline characteristics of patients with PDAC.

**Characteristics**	**N(%)**
Gender	
Male	22 (47.8%)
Female	24 (52.8%)
Age	
<60	13 (28.3%)
≥60	33 (71.7%)
CA19-9	
≥27U/ml	40 (87.0%)
<27U/ml	6 (13.0%)
Stage	
I	15 (32.6%)
II	21 (45.7%)
III	3 (6.5%)
IV	7 (15.2%)
Metabonomics	
yes	27 (58.7%)
no	19 (41.3%)

### RIOK3 promotes arginine uptake in PDAC cells by upregulating SLC7A2 expression

**Figure 2:**
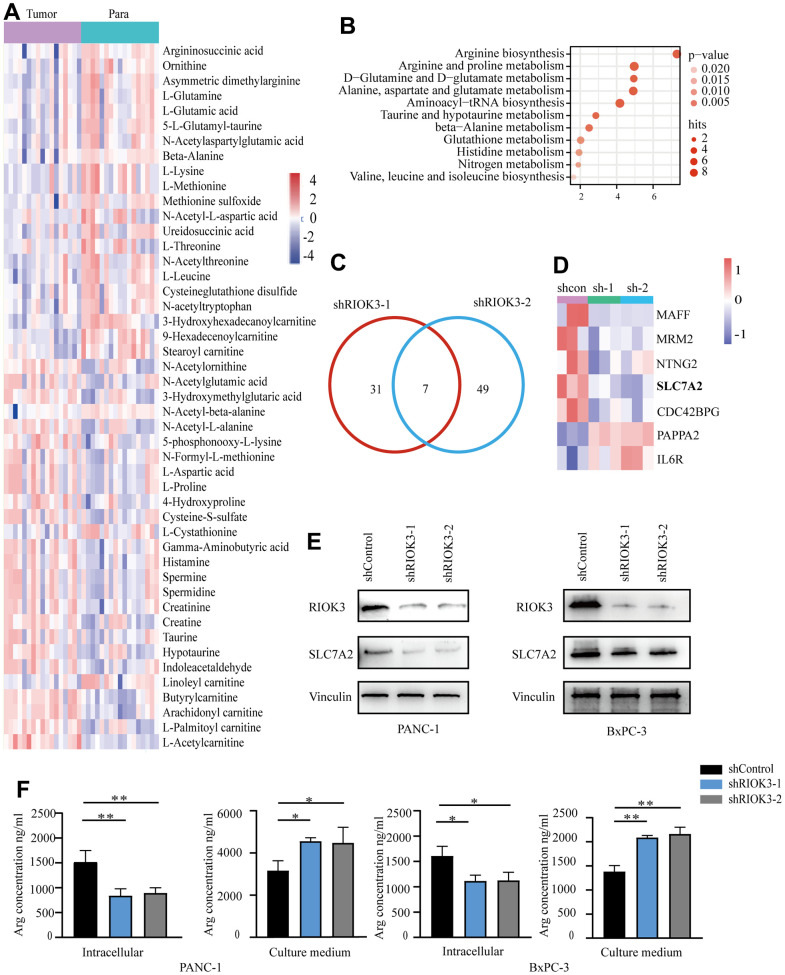
**Metabolomic and transcriptional profiling reveals RIOK3 promotes arginine uptake in PDAC cells by upregulating SLC7A2 expression.** (**A**) Heatmap of the differential metabolites of high-RIOK3 expression PDAC tissues compared with the paired adjacent normal tissues based on nontargeted metabolomic analysis (| Log2FC |>1, p<0.05). Blue represents the decreasing trend, and red represents the increasing trend. Paired Samples Wilcoxon test. (**B**) KEGG pathway enrichment analysis of the 47 differential metabolites. (https://www.metaboanalyst.ca/). (**C**) Venn diagram summarizing that 7 mRNAs were detected in the two independent RIOK3-knockdown cells (| Log2FC |>1, p<0.05). (**D**) Heatmap of 7 mRNAs that were detected in the two independent RIOK3-knockdown cells (| Log2FC |>1, p<0.05). (**E**) Western blot analysis of RIOK3 and SLC7A2 in shRIOK3-1/2 cells (PANC-1 and BxPC-3 cells, respectively). One-way ANOVA test. (**F**) The content of Arg was analyzed in the intracellular and culture medium of shRIOK3-1/2 cells (PANC-1 and BxPC-3 cells, respectively). One-way ANOVA test. All experiments were performed independently at least three times. The star (*) symbol denotes the level of statistical significance: * p < 0.05, ** p < 0.01, *** p < 0.001, ns no significance.

Abnormal arginine metabolism is one of the characteristics of metabolic reprogramming in PDAC [12]. Subsequently, we selected 27 pairs of PDAC and adjacent normal fresh tissues in the above microarray sample set for metabolomic analysis. The results showed that, compared with the paired adjacent normal tissues, there were 47 different metabolites in PDAC tissues with high RIOK3 expression (n=17) (Figure 2A), and pathway enrichment analysis suggested that these differential metabolites were associated with arginine synthesis and arginine and proline metabolism (Figure 2B), whereas only 28 differential metabolites were found in cancer tissues with low RIOK3 expression (n=10) (Supplementary Figure 1A), and the pathway enrichment analysis did not reveal any pathway associated with arginine metabolism (Supplementary Figure 1B). To further clarify whether the aberrant expression of RIOK3 is associated with arginine metabolism, we constructed PANC-1cell lines with stable knockdown of RIOK3 (shRIOK3-1 and shRIOK3-2) and performed metabolomic analysis. We found 33 different metabolites in 2 different RIOK3 knockdown cell lines (Supplementary Figure 1C). Pathway enrichment analysis showed that these different metabolites were linked to arginine biosynthesis and arginine and proline metabolism (Supplementary Figure 1D). Overall, we found that RIOK3 was associated with arginine metabolism in PDAC.

To further explore the specific mechanism of RIOK3 regulation of arginine metabolism, we performed high-throughput transcriptome analysis on the above cell lines. Following RIOK3 knockdown, 7 genes were significantly altered in the two independent RIOK3 knockdown cell lines (Figure 2C). Among them, the arginine transporter protein SLC7A2 was significantly downregulated with RIOK3 knockdown (Figure 2D). SLC7A2 is an inducible transporter of the semi-essential amino acid arginine, which is one of the main carriers for the transport of arginine from the extracellular environment. Subsequent western blot analysis also confirmed that, with the knockdown of RIOK3, the protein of SLC7A2 was similarly significantly downregulated in both PANC-1 and BxPC-3 cell lines (Figure 2E). Based on the results of metabolomic and transcriptomic analyses, we hypothesized that RIOK3 could increase arginine uptake mediated by SLC7A2. Subsequently, we quantified the arginine content in both the culture medium and the cells, and found that when RIOK3 was knockdown, the arginine content significantly reduced in cells, and increased in the cell culture medium (Figure 2F). Overall, our results revealed that RIOK3 was significantly associated with arginine metabolism in PDAC and that RIOK3 may promote arginine uptake through upregulation of SLC7A2 expression.

### RIOK3 activates mTORC1 by promoting the uptake of arginine

**Figure 3:**
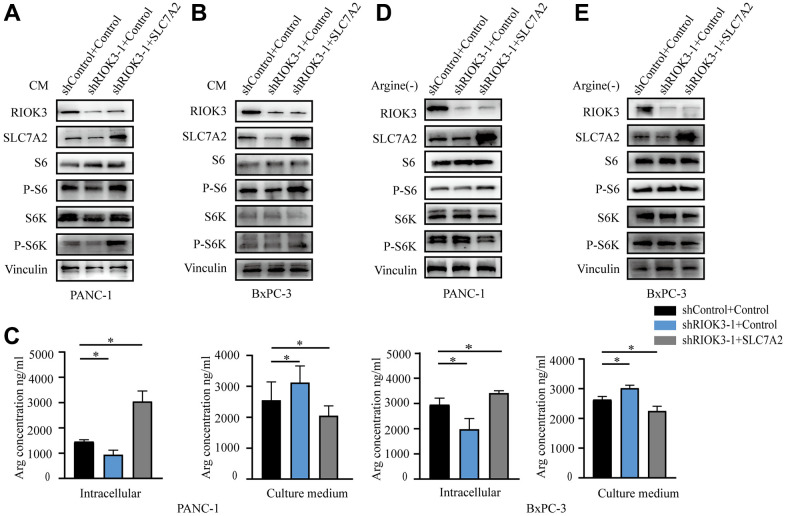
**RIOK3 promotes the activation of the mTORC1 pathway via SLC7A2.** (**A**, **B**) Western blot analysis of RIOK3, SLC7A2, S6, P-S6, S6K and P-S6K in PANC-1 and BxPC-3 cells transduced with shRIOK3-1 alone or in combination with SLC7A2 under conventional culture conditions. (**C**) The content of Arg was analyzed in the intracellular and culture medium of PANC-1 and BxPC-3 cells transduced with shRIOK3-1 alone or in combination with SLC7A2 under conventional culture conditions. One-way ANOVA test. (**D**, **E**) Western blot analysis of RIOK3, SLC7A2, S6, P-S6, S6K and P-S6K in PANC-1 and BxPC-3 cells transduced with shRIOK3-1 alone or in combination with SLC7A2 under arginine-deficient culture conditions. All experiments were performed independently at least three times. The star (*) symbol denotes the level of statistical significance: * p < 0.05, ** p < 0.01, *** p < 0.001, ns no significance.

To confirm the critical role of SLC7A2 in arginine uptake involved in RIOK3, we overexpressed SLC7A2 in cell lines with knockdown of RIOK3 (Figure 3A, 3B). A subsequent assay of arginine content revealed that when RIOK3 knocked down, the reintroduction of SLC7A2 increased intracellular arginine content and reduced arginine content in the culture medium (Figure 3C).

Aberrant activation of mTORC1 protein kinase in cancer is associated with accelerated cell proliferation and anabolism [21]. Various amino acids, including glutamine and arginine, are involved in regulating the activation of mTORC1 [13, 22, 23]. To clarify whether RIOK3 activates mTORC1 by promoting the transport of arginine, we examined the phosphorylation of S6 and S6 kinase (S6K) under normal and arginine-deficient culture conditions, respectively. First, the phosphorylation of S6 and S6K was significantly reduced with the knockdown of RIOK3 under conventional culture conditions. The reintroduction of SLC7A2 significantly promoted the activation of S6 and S6K (Figure 3A, 3B). However, no trend was observed for the activation of S6 and S6K culture under arginine-deficient culture conditions (Figure 3D, 3E). Based on these results, we revealed that RIOK3 knockdown inhibits mTORC1 activation by suppressing SLC7A2-mediated arginine uptake.

### RIOK3 promotes invasion and metastasis of PDAC cells via SLC7A2

**Figure 4:**
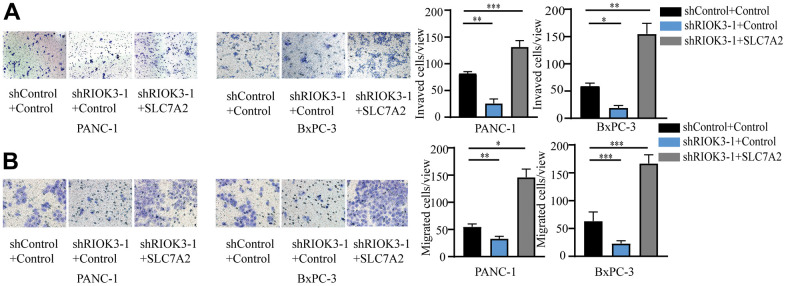
**RIOK3 promotes invasion and migration of PDAC cells via SLC7A2.** (**A**, **B**) The invasion (**A**) and migration (**B**) abilities of the above cell lines in E were determined using the migration and invasion assays, respectively. One-way ANOVA test. All experiments were performed independently at least three times. The star (*) symbol denotes the level of statistical significance: * p < 0.05, ** p < 0.01, *** p < 0.001, ns no significance.

Our previous study confirmed that RIOK3 plays an important role in the invasive metastasis of PDAC. To further clarify whether SLC7A2 is involved in this process, we performed invasive and metastasis assays using the above cell lines. We found that RIOK3 knockdown significantly inhibited the invasion and metastasis of PDAC cells, and the reintroduction of SLC7A2 was indeed able to compensate for the reduced invasion and metastasis caused by RIOK3 knockdown (Figure 4A, 4B). Overall, SLC7A2 plays a key role in RIOK3-mediated invasion and metastasis of PDAC cells.

### High RIOK3 expression combined with Treg infiltration was significantly associated with poor prognosis in PDAC patients

**Figure 5:**
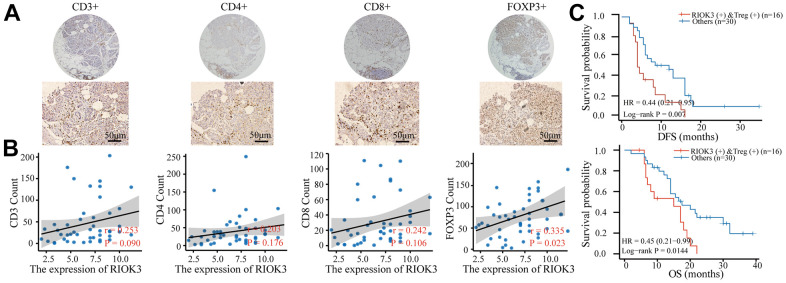
**Patients with RIOK3 and Treg cell positivity have a worse prognosis.** (**A**) Representative IHC staining of CD3, CD4, CD8, and FOXP3 in PDAC tissues (n=46). The cut-off value of immune cells was median. (**B**) The association between RIOK3 expression and the number of CD3, CD4, CD8, and FOXP3 cells in PDAC (|r|>0.2 and p<0.05). R represents the Pearson correlation coefficient. (**C**) Kaplan–Meier curves of DFS and OS analysis according to RIOK3 and Treg cell levels in PDAC patients (n=46, 16 RIOK3-high+Treg-high and 30 others).

The content of arginine in the PDAC microenvironment is closely related to infiltrating lymphocytes [24]. We subsequently explored the correlation between RIOK3 and infiltrating lymphocytes in PDAC tissues. First, 46 PDAC tissues were stained with IHC for CD3, CD4, CD8, and FOXP3 and counted. Subsequent correlation analysis showed that RIOK3 was positively correlated with Treg cells (r=0.335, P=0.023), while there was no significant correlation with CD3+, CD4+, and CD8+ T cells (Figure 5A, 5B). In addition, we found that RIOK3 high combined with Treg high patients had significantly shorter DFS (12.0 vs. 4.2 months, HR=0.44, 95% CI 0.21-0.95, p=0.007) and OS (17.5 vs. 15.5 months, HR=0.45, 95% CI 0.21-0.99, p=0.0144) (Figure 5C) than other patients. These results suggest that high expression of RIOK3 and Treg infiltration are significantly associated with poor prognosis in PDAC patients.

## DISCUSSION

**Figure 6:**
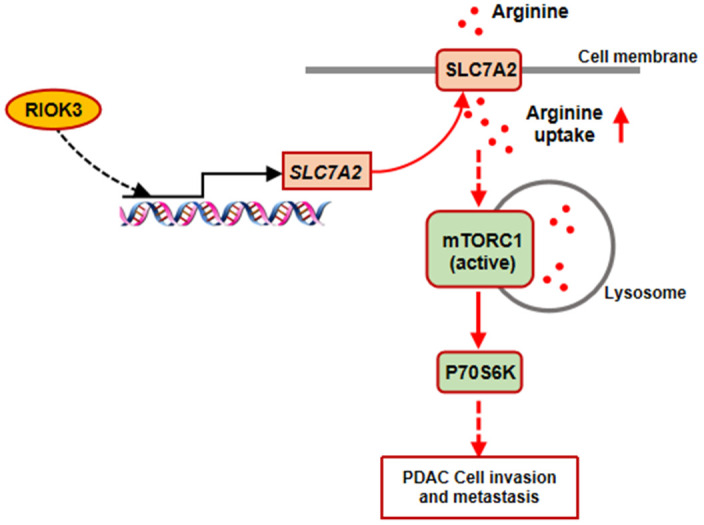
**Diagram of the proposed mechanism.** RIOK3 promotes mTORC1 activation by facilitating SLC7A2-mediated arginine uptake in PDAC.

In this study, we identified that SLC7A2-mediated acceleration of arginine uptake is a key process by which RIOK3 promotes mTORC1 activation in PDAC cells (Figure 6). In addition, we found that High RIOK3 expression combined with Treg infiltration was significantly associated with poor prognosis in PDAC patients.

PDAC is one of the most lethal malignancies, with an extremely poor prognosis. Metabolic reprogramming is a novel feature of cancer. In addition to enhanced glycolysis, amino acid metabolic reprogramming has been increasingly emphasized. ASS1 converts citrulline to arginosuccinate and is the rate-limiting enzyme for arginine synthesis [25]. It is normally highly expressed in normal tissues but is lost in expression in a range of tumor types, including PDAC [26]. The deficiency of ASS1 expression makes PDAC potentially more dependent on exogenous arginine supplementation, and arginine deprivation has emerged as a promising therapeutic option. Additionally, due to the enhanced metabolism, tumor cells take up a larger amount of nutrients than normal cells, a process that requires specific organic material transporters. SLC7A2 is an inducible transporter of the semi-essential amino acid arginine, which is one of the main carriers for the transport of arginine from the extracellular environment [27, 28]. In this study, we found that the high expression of RIOK3 in PDAC cells and tissues was significantly correlated with arginine metabolism by metabolome and transcriptome analysis. Specifically, RIOK3 promoted arginine uptake in PDAC cells through upregulation of SLC7A2 expression.

Intracellular arginine was associated with mTORC1 activation. Chantranupong et al. found that mTORC1 can be activated by arginine sensing, which is mediated by mTORC1 subunit 1 (CASTOR1) and solute carrier family 38 member 9 (SLC38A9) [13, 14]. In addition, arginine regulates lysosomal concentrations of various essential amino acids and increases phosphorylation of P70 S6K. Arginine binding to mTORC1 activation releases essential amino acids from lysosomes and promotes the growth of PDAC cells, which can obtain large amounts of amino acids through massive cytosolization of extracellular proteins [15, 16]. We observed that knockdown of RIOK3 significantly inhibited S6K activation, and reintroduction of SLC7A2 rescued S6K activation. However, these changes were not observed in arginine-deficient cultures. In addition, reintroduction of SLC7A2 also rescued the downregulation of invasion and metastasis mediated by RIOK3 knockdown. These results further confirm that a SLC7A2-mediated increase in arginine uptake plays a key role in RIOK3-promoted mTORC1 activation.

PDAC is a low-immunogenic tumor with a highly immunosuppressive microenvironment. Treg cells are considered one of the master regulatory cells, which not only secrete cytokines that promote the proliferation of tumor cells, but also play an indispensable role in inducing new angiogenesis and metastasis [29, 30]. It has been found that arginine supplementation may enhance antitumor effects by enhancing immune function. Arginine supplementation has been found to stimulate T cell and natural killer cell activity and promote the production of proinflammatory cytokines [31, 32]. Our study found that RIOK3 overexpression significantly promoted arginine uptake in PDAC cells and resulted in a decrease in extracellular arginine content. Based on this result, we hypothesized that RIOK3 might be associated with the number of lymphocytes in PDAC. Subsequent IHC experiments revealed that high expression of RIOK3 was associated with the number of Treg cells in PDAC tissues. In addition, RIOK3-high combined with Treg-high patients had significantly shorter DFS and OS than other patients.

Overall, this study concluded that RIOK3 is one of the key proteins that promote arginine uptake in PDAC cells and revealed that SLC7A2-mediated acceleration of arginine transport is a critical link in this process. Furthermore, an increase in arginine uptake mediated by RIOK3 is one of the important factors for mTORC1 activation in PDAC cells. In conclusion, RIOK3 is expected to be an exciting therapeutic target, and its expression may lead to a breakthrough in the prediction of efficacy of immunotherapy in PDAC.

## MATERIALS AND METHODS

### Clinical specimens

46 pairs of PDAC samples were obtained from the first affiliated hospital of Dalian Medical University (Dalian, China), and all samples were collected with the informed consent of the patients and stored in the biobank. All human samples included in the study were handled in accordance with the tenets of the Declaration of Helsinki.

### Immunohistochemistry

IHC staining procedures were performed according to the instructions of the IHC Kit (KIHC-5; ProteinTech, Wuhan, China). RIOK3 antibody (ab118483 1: 100; Abcam) CD3 (1:150, Abcam, ab16669), CD4 (1:200, CST, 48274) and CD8 (1:30, CST, 85336) were used as the primary antibody. EDTA and citrate solution were used for antigen retrieval depending on the antibody instructions. The H-score (H-SCORE=∑(pi×i) = (percentage of weak intensity×1) + (percentage of moderate intensity×2) + (percentage of strong intensity×3)) was used to assess the RIOK3 staining intensity. And the positively stained cells around the tumor cells were evaluated with a microscope in three randomly selected fields at a magnification of × 400 and the average was calculated as the number of CD3, CD4, CD8, and Treg cells.

### Cell culture

The human PDAC cell lines PANC-1 and BxPC-3 cells were obtained from the Cell Bank of the Chinese Academy of Sciences (Shanghai, China), HEK293T cells were stored in the lab, and PANC-1 and HEK293T cells were cultured in DMEM (GIBCO, USA), BxPC-3 cells were cultured in RPMI-1640 (GIBCO, USA). Cell lines were maintained in culture supplemented with 10% FBS (GIBCO, USA) and 1% penicillin/streptomycin (Thermo Fisher, USA) at 37° C with 5% CO_2_ in a humidified incubator (Thermo Fisher, USA).

### Plasmids

SLC7A2 cDNA (HG11124-M, Sinobiological, China) were subcloned into pBoBi expression vectors which were cut by XhoI and BamHI. The expression vectors were produced utilizing Exonuclease III (M0206L, NEB, USA). Transfection was performed with Lipofectamine 2000 (29223-1-AP 1:1000, Proteintech, China).

Lentiviral shRNAs were cloned in pLKO.1 within the AgeI/EcoRI sites at the 3’end of the human U6 promoter. The targeted sequences were:

shRIOK3-1: 5’-CTGTTGTCTTTCATGCATATG-3’

shRIOK3-2: 5’-GTTGCGTCTTTCCTTGAATAT-3’

### Construction of stably transfected cell lines

To package the virus, we seeded HEK-293T cells in a 10 cm dish the day before at 60% density. Polyethyleneimine Linear was used to co-transfect with the package plasmid psPAX2 and pVSVG. Cells needed nutritional support with 2ml supplemental medium after 24 hours. Then, 48 hours after transfection, condition media containing recombinant lentiviruses was collected and filtered with a 0.22-μm filter (Merck Millipore, Billerica, MA, USA). Supernatants from these samples were immediately administered to target cells along with Polybrene (Beyotime, Shanghai, China) at a final concentration of 10 mg/ml, and the supernatants were incubated with the cells for 12 hours. The stable cell lines were maintained with 2 ug/mL puromycin (Beyotime, Shanghai, China).

### LC-MS-based metabolomic analyses

### Cell metabolites

The experiments were performed as described previously [33]. Briefly, cell culture plates were washed with PBS and snap-frozen in liquid nitrogen and stored at -80° C. Then, added an internal standard mixture of carnitine C5:0-d2, lysophosphatidylcholine (LPC) 14:0 sn-1, LPC 18:1 sn-1, and liquid phase exfoliation (LPE) 18:1 sn-1 were added to the culture plate. Then we vortexed and centrifugated, and the supernatant was stored at -80° C. We analyzed the samples with a UPLC (Waters Corp., Milford, MA, USA) coupled to a Triple Q Exactive Mass Spectrometer (Thermo Fisher Scientific USA).

### Tissue metabolites

The detailed methods were as described before [34]. Briefly, 20 mg of the tissue sample were taken. After grinding and vortexed, we get lipid extract transferred from the upper layer and the polar extract from the lower layer. The sample was done on an Ultimate 3000 ultra-high performance liquid chromatography and Q Exactive quadrupole-Orbitrap high-resolution mass spectrometer (Thermo FisherScientific, USA).

### RNA isolation and RNA sequencing

We used our previous study RNA Sequencing results and repeated the experiment as described previously [20]. The differential gene was considered as p <0.05 and |log2(FC)|>1.

### Western blot

Western blot analysis was performed with standard methods [20]. The following antibodies and reagents were used: anti-RIOK3 (A305-601A-T, Thermo Fisher, USA), anti-SLC7A2 (A14574, Abclonal, China), P70S6K (14485-1-AP, Proteintech, China), phospho-P70S6K (28735-1-AP, Proteintech, China), PS6 (14823-1-AP, Proteintech, China) and Phospho-PS6 (Ser235/236) (14823-1-AP, Proteintech, China). HRP Goat Anti-Mouse IgG (H+L) (AS003, Abclonal, China), HRP Goat Anti-Rabbit IgG (H+L) (AS014, Abclonal, China). In brief, cells were harvested by radioimmunoprecipitation assay (RIPA) buffer. We determined protein concentrations with a protein quantitation kit (Bio-Rad, Hercules, CA), according to the standard methods. 20 ug protein of each sample was separated by sodium dodecyl sulfate-polyacrylamide gel electrophoresis (SDS-PAGE). The primary antibody was probed for blots overnight at 4° C. The secondary antibody was incubated for 1 h at room temperature.

### KEGG pathway enrichment analysis

Significantly different metabolites (p<0.05 |log2(FC)|>0.3) of the RIOK3 stable knockdown PANC1 cells and significantly different metabolites (p<0.05 |log2(FC)|>1) of PDAC tissues and paired adjacent normal tissues were subjected to KEGG pathway enrichment analysis using Metaboanalyst 5.0 (https://www.metaboanalyst.ca/) in the LC-MS data.

### Arginine concentration

We measured arginine content according to the human Arg Elisa kit instructions with standard methods (Jingmei, Yancheng, China).

### Migration and invasion assays

We measured the vitro migration and invasion assays in Transwell chambers (8-uM pore size; Corning, NY, USA). After transfection, cells were seeded in the upper chamber at a density of 4 × 105 with complete culture medium. The lower chamber supplied with a culture medium without 10% FBS. Invasion assay need to lay the matrix glue (AWM, Shanghai, China) in the upper chamber. Following a 36h (for PANC-1 cells) or 24h (for BxPC-3 cells) incubation, we fixed for staining invaded cells to the bottom in crystal violet solution. Three random fields of each membrane were viewed and the number of cells on the underside was counted under the inverted microscope.

### Statistical analysis

We used the R studio and R packages (version 3.6) to analyze PDAC tissue RNA-seq data from TCGA in HTSeq-FPKM format (https://portal.gdc.cancer.gov/). The mean value of the two groups was compared using paired Student’s t-test and paired Wilcoxon test. One-way ANOVA test was used to compare above two groups mean value, while the Kaplan–Meier test was used to calculate the differences in survival. Metabolomics data need inter standard correction. All of the relative protein expression was normalized by ImageJ (version no.: 1.8.0_112; https://imagej.nih.gov/ij/). Bars and error represent the mean ± standard deviation (SD) of replicate measurements. *p<0.05, **p<0.01 and ***p<0.001 indicate statistical significance. The SPSS 18.0 software package (SPSS, Inc., Chicago, IL, USA) was used for statistical analysis. The heatmap and bar plots were conducted on R studio (version 3.6). Kaplan–Meier curves were conducted on GraphPad Prism 8.0. All of the p-values involved in this study were two-tailed probabilities. The difference was statistically significant for p <0.05.

### Abbreviations

PDAC: Pancreatic ductal adenocarcinoma; RIOK3: Rio Kinase 3; SLC7A2: solute carrier family 7 member 2; Treg: Regulatory T; mTOR: mechanistic target of rapamycin; WHO: World Health Organization; NO: nitric oxide; mTORC1: mechanistic target of rapamycin complex 1; ASS1: Arginosuccinate synthase; TCGA: The Cancer Genome Atlas; GTEx: Genotype-Tissue Expression; ROC: Receiver operating characteristic; AUC: area under curve; DFS: disease free survival; OS: overall survival; RPS6: Ribosomal Protein S6; S6K: Ribosomal S6 kinase; SLC38A9: solute carrier family 38 member 9; P-S6K: P70 S6 kinase 1.

## Supplementary Material

Supplementary Figure 1
