# Supplementary material for: RIOK3 promotes mTORC1 activation by facilitating SLC7A2-mediated arginine uptake in pancreatic ductal adenocarcinoma
Source: Aging (Albany NY). 2023 Feb 24;15(4):1039–51. doi: 10.18632/aging.204528 (PMC10008507; doi:10.18632/aging.204528)
Supplement: Supplementary Figure 1 [file aging-15-204528-s001.pdf]

SUPPLEMENTARY FIGURE

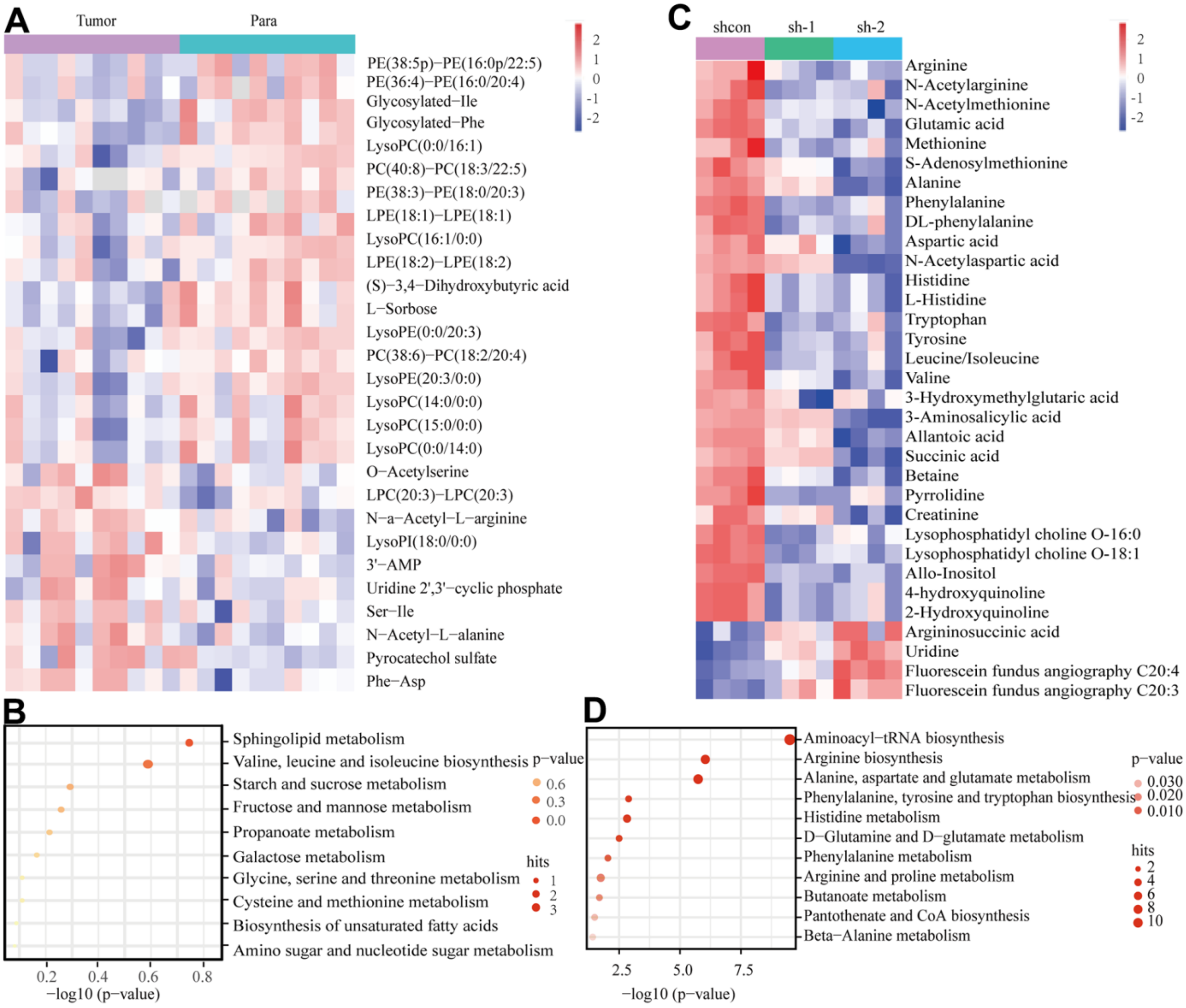

Supplementary Figure 1. (**A**) Heatmap of the differential metabolites of low-RIOK3 expression-PDAC tissues compared with the paired adjacent normal tissues based on nontargeted metabolomic analysis (| Log2FC |>1, p<0.05). Blue represents the decreasing trend, and red represents the increasing trend. Paired Samples Wilcoxon test. (**B**) KEGG pathway enrichment analysis of the differential metabolites in A. (**C**) Heatmap of the differential metabolites related to RIOK3 knockdown PDAC cells based on nontargeted metabolomic analysis (| Log2FC |>0.3, p<0.05). Paired Samples Wilcoxon test. (**D**) KEGG pathway enrichment analysis of the differential metabolites in C.
